# A randomized crossover, pilot study examining the effects of a normal protein vs. high protein breakfast on food cravings and reward signals in overweight/obese “breakfast skipping”, late-adolescent girls

**DOI:** 10.1186/1475-2891-13-80

**Published:** 2014-08-06

**Authors:** Heather A Hoertel, Matthew J Will, Heather J Leidy

**Affiliations:** Department of Nutrition & Exercise Physiology, School of Medicine, University of Missouri, 207 Gwynn Hall, Columbia, MO 65211 USA; Department of Psychological Sciences, University of Missouri, Columbia, MO 65211 USA

**Keywords:** High protein breakfast, Food reward, Food cravings, Homovanillic acid, Dopamine

## Abstract

**Background:**

This pilot study examined whether the addition of a normal protein (NP) vs. high protein (HP) breakfast leads to alterations in food cravings and plasma homovanillic acid (HVA), which is an index of central dopamine production, in overweight/obese ‘breakfast skipping’ late-adolescent young women.

**Methods:**

A randomized crossover design was incorporated in which 20 girls (age 19 ± 1 y; BMI 28.6 ± 0.7 kg/m^2^) consumed 350 kcal NP (13 g protein) breakfast meals, 350 kcal HP (35 g protein) breakfast meals, or continued breakfast skipping (BS) for 6 consecutive days/pattern. On day 7 of each pattern, a 4 h testing day was completed including the consumption of breakfast (or no breakfast) followed by food craving questionnaires and blood sampling for HVA concentrations throughout the morning.

**Results:**

Both breakfast meals reduced post-meal cravings for sweet and savory foods and increased HVA concentrations vs. BS (all, p < 0.05). Between breakfast meals, the HP breakfast tended to elicit greater reductions in post-meal savory cravings vs. NP (p = 0.08) and tended to elicit sustained increases in HVA concentrations prior to lunch vs. NP (p = 0.09). Lastly, HVA concentrations were positively correlated with the protein content at breakfast (r: 0.340; p < 0.03).

**Conclusions:**

Collectively, these findings suggest that the addition of breakfast reduces post-meal food cravings and increases homovanillic acid concentrations in overweight/obese young people with higher protein versions eliciting greater responses.

## Introduction

Adolescent obesity continues to be a growing public health concern in the United States, affecting the lives of over 25 million young people [1, 2]. Since approximately 36% of adolescents are overweight or obese, and up to 80% will likely become overweight adults, it is essential to focus on young people to prevent this epidemic from perpetuating into future generations [2, 3]. Although the etiology of obesity is multifactorial, several behavioral and environmental factors have been implicated. Of particular interest is the increasingly common dietary habit of breakfast skipping, which has closely mirrored the rise in obesity [4, 5]. Specifically, reduced breakfast frequency (i.e., breakfast skipping) is inversely associated with increased BMI, weight gain, and obesity in young people [4–6]. However, due to the lack of long-term randomized controlled trials, a causal link between breakfast skipping and obesity has not been substantially identified [7, 8].

We previously found that skipping the morning meal leads to increased perceived hunger and reduced perceived fullness (satiety), and greater energy intake at subsequent eating occasions compared to eating breakfast, particularly one rich in dietary protein [9, 10]. Potential mechanisms of action include the marked elevations in the hunger-hormone ghrelin and lower concentrations of the satiety hormone PYY [10]. Since most Americans eat (or stop eating) for reasons other than ‘physiological’ hunger or fullness [11], it is critical to extend these findings to explore the signals controlling food cravings and food reward.

Dopamine is a powerful neural signal implicated in the regulation of food intake by stimulating reward-driven eating behavior [12–14]. A negative correlation has been reported between dopamine and BMI with obese individuals displaying a blunted dopamine response in proportion to BMI [15–17]. In obese animal models, central dopamine activity is reduced and is accompanied by elevated preference for highly palatable, energy dense foods compared to normal weight controls [18]. However, treatment with dopamine agonists reverses the excess body weight and obesity [17]. Thus, these data suggest that strategies to stimulate dopamine activity may lead to significant improvements in obesity.

In general, dopamine is secreted in response to tasting highly palatable foods and evokes feelings of enhanced food reward, pleasure, and well-being [19–21]. Although much of the literature has focused on dietary fat and sugar as key stimulants of food reward [22, 23], dietary protein has also been speculated to elicit similar reward responses [19]. Data from our lab and others support the benefits of increased protein consumption for improved appetite control, satiety, and body weight management [24]. We would like to extend these findings to examine the role of increased dietary protein at breakfast on signals controlling food reward.

However, since central dopamine is unable to cross the blood–brain barrier [25], it is challenging to appropriately measure its activity. One alternative is to measure dopamine metabolites in body fluids, particularly plasma [26]. The most predominant metabolite is plasma homovanillic acid (HVA) [27, 28] which is strongly correlated with central dopaminergic activity [29–31] and has been suggested to be the most appropriate indicator of central dopamine activity [27].

Thus, the primary purpose of this study was to assess whether the daily addition of a normal vs. protein-rich breakfast alters food cravings and HVA responses throughout the morning in overweight/obese ‘breakfast skipping’ late-adolescent young women. In addition, the relationship between HVA and dietary protein and food cravings were also identified.

## Materials and methods

### Experimental design

This study was part of a larger study designed to examine the beneficial effects of a higher-protein breakfast on the appetitive, hormonal, and neural signals controlling energy intake regulation in overweight/obese, “breakfast-skipping”, late-adolescent girls [10]. Sixteen overweight and obese ‘breakfast skipping’ late-adolescent young women participated in the following randomized crossover-design breakfast study. The participants randomly completed the following breakfast patterns at home for 6 days: 1) Breakfast Skipping (BS); 2) Consumption of Normal Protein (NP) breakfast meals; and 3) Consumption of Higher Protein (HP) breakfast meals. On day 7 of each pattern, the participants came to the University of Missouri Brain Imaging Center (MU-BIC) in the morning to complete the respective 4-h testing day. The participants began the testing day by either skipping breakfast or consuming their respective breakfast meal. Blood samples and assessments of food cravings were completed at specific times throughout the morning. There was at least a 7-day washout period between each pattern. The main trial was registered at clinicaltrials.gov as NCT01192100.

### Study participants

Late-adolescent young women were recruited from the Columbia, MO area through advertisements, flyers, and parent-received University of Missouri listservs to participate in the study. Eligibility was determined through the following inclusion criteria: 1) age range of 13–20 y; 2) overweight to obese (BMI: 25–34.9 kg/m^2^); 3) no metabolic or neurological diseases or other health complications; 4) no clinical diagnosis of an eating disorder; 5) not currently or previously on a weight loss or other special diet in the past 6 months; 6) documented regular menstrual cycles between 21–36 days in duration for the past 6 months; and 7) infrequent breakfast consumer (i.e., ≤ 2 breakfast occasions/wk).

One-hundred and forty-seven teens were interested in participating in the study. Twenty-five met the screening criteria, had three available Saturdays to complete the 4 h testing days, and began the study. Sixteen completed all study procedures. The participants were, on average, 19 ± 1 y of age, BMI: 28.6 ± 0.7 g/m^2^, and skipped breakfast an average of 6 ± 1 times/week. All participants and their parents (if participant is <18 y of age) were informed of the study purpose, procedures, and risks and signed the consent/assent forms. The study was approved by the University of Missouri Health Sciences Institutional Review Board. The participants received a stipend for completing all study procedures.

### Breakfast patterns

The participants completed each of the three breakfast patterns for seven consecutive days. For the BS pattern, the participants continued to follow their habitual practice of skipping breakfast and completed the Day 7 testing day accordingly. For the NP and HP patterns, the participants were provided with specific breakfast meals and asked to consume these at home (before school) between 7:00 am-9:30 am for 6 days. Throughout this period, the participants were permitted to eat ad libitum throughout the remainder of each day. On Day 7, they completed the respective testing day. There was a 7-day washout period in between each of the breakfast patterns in which all participants returned to their previous ‘breakfast skipping’ behavior. For additional information, please see [10].

### Breakfast meals

The breakfast energy intake for the breakfast meals was comprised of 18% of the total energy intake (350 kcal) as estimated from the energy expenditure equations specific for adolescents [32]. The macronutrient composition of the NP breakfast contained 15% protein (13 g of dietary protein), 65% CHO, and 20% fat whereas the HP breakfast contained 40% protein (35 g of protein, 60% of protein from egg and beef sources), 40% CHO, and 20% fat. In addition to being matched for fat content, the breakfast meals were similar in energy density, dietary fiber, and sugar content. (See Table 1). In addition, the meals were matched for palatability and sensory/hedonic factors (data not shown).

**Table 1:** Breakfast characteristics*

	Breakfast skipping (BS)	Normal protein (NP)	High protein (HP)
Energy content	0	350	350
Mass (g)	0	270	265
Energy density (kcal/g)	0	1.33 ± 0.01	1.37 ± 0.03
Total protein (g) (% of meal)	0	13.0 (15%)	35.1 (40%)
Egg (g)	0	0	11.0
Beef (g)	0	0	11.0
Dairy (g)	0	7.0	7.0
Gluten (g)	0	6.0	6.0
Tyrosine (g)	0	0.55	1.34
Total carbohydrate (g) (% of meal)	0	57.0 (65%)	35.1 (40%)
Sugar (g)	0	18.0	18.0
Fiber (g)	0	6.1	6.1
Total fat (g) (% of meal)	0	7.8 (20%)	7.8 (20%)

*Excluding the 8 oz. water that was provided along with the breakfast meal.

### Testing day procedures

On Day 7 of each breakfast pattern, the participants reported to the MU-BIC research facility between 6–9 am after an overnight fast to complete the 4 h testing day. Each participant was seated in a reclining chair and became familiarized with the testing day procedures. Prior to breakfast (-30 min), a catheter was inserted into the antecubital vein of the non-dominate arm and kept patent by saline drip throughout the remainder of the testing day. At time -15 min, a baseline (fasting) blood sample was drawn and a set of computerized food craving questionnaires were completed. At time 0 min, a meal including water was provided during the NP and HP days and only water during the BS day. The participants were instructed to consume the meal and/or water within 30 min, making sure to eat at a rate that was consistent with their habitual, normal eating habit.

Validated, computerized questionnaires, assessing cravings for sweet or savory foods were completed at times -30, +0, 30, 60, 120, 180, and 240 min [33]. The questionnaires contained visual analog scales incorporating a 100 mm horizontal line rating scale for each response. The questions were worded as “how strong is your feeling of” with anchors of “not all” to “extremely”. The Adaptive Visual Analog Scale Software was used for data collection (Neurobehavioral Research Laboratory and Clinic; San Antonio, TX).

The participants remained in the reclining chair for the duration of testing day but were permitted to do school work, work on their laptop computer, and use the restroom when necessary.

Blood samples (4 ml/sample) were also collected at times +0, 30, 60, 120, 180, and 240 min. The samples were collected in test tubes containing EDTA (ethylenediaminetetra-acetic acid) and were place in ice for 10 min. Within 10 min of collection, the samples were centrifuged at -4°C, 3500 rpm for 10 min. The plasma was separated and stored in microcentrifuge tubes at -80°C for future analysis. Plasma homovanillic acid (HVA), the primary dopamine metabolite, was measured with an enzyme-linked immunosorbent assay (Cat#: HVA34-K01; Eagle Biosciences, Nashua, NH). The assay sensitivity is 0.035 – 0.125 μg/ml. The intra and inter-assay coefficients of variation are 6.0 and 8.0%, respectively.

## Data and statistical analyses

Power analyses for the food craving assessments were performed to identify appropriate sample size. The effect size of the protein-related changes in sweet cravings and savory cravings following normal vs. high protein breakfast meals were determined from Belza et al. [34]. The effect sizes were 0.54 and 1.06 for sweet and savory cravings, respectively. With these effect sizes, an n = 16 at α = 0.05 would provide .80% power to detect differences between breakfast treatments. Since there are no known human studies published to date with post-prandial HVA concentrations, observed power (OP) was performed and reported.

Summary statistics (sample means and/or 4-h incremental area under the curve (AUC)) were computed for all study outcomes. A repeated-measures ANOVA was performed to determine main effects of time, treatment, and interactions for food cravings and HVA concentration outcomes. When main effects were detected, post-hoc analyses including pairwise comparisons were performed using Least Significant Difference procedures to identify differences among treatments. Pearson correlations were conducted to determine associations between HVA concentrations and the following factors: dietary protein and tyrosine concentrations (at breakfast) and food cravings. Data was expressed as mean ± SEM. Analyses were conducted with the latest version of SPSS (Chicago, IL). P < 0.05 was considered statistically significant.

## Results

**Figure 1:**
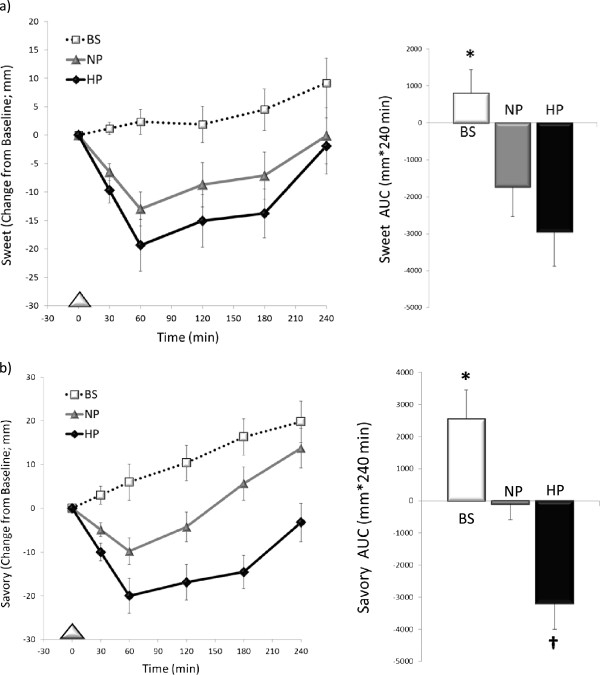
**Food craving responses. a)** Sweet responses over the 4 h post-breakfast period comparing breakfast skipping (BS), normal protein (NP), and high protein (HP) patterns; *Post-hoc analyses, BS vs. NP & HP, p < 0.05; Data is expressed as mean ± SEM. **b)** Savory responses over the 4 h post-breakfast period comparing breakfast skipping (BS), normal protein (NP), and high protein (HP) patterns; *Post-hoc analyses, BS vs. NP & HP, p < 0.05; †NP vs. HP, p = 0.08; Data is expressed as mean ± SEM.

**Figure 2:**
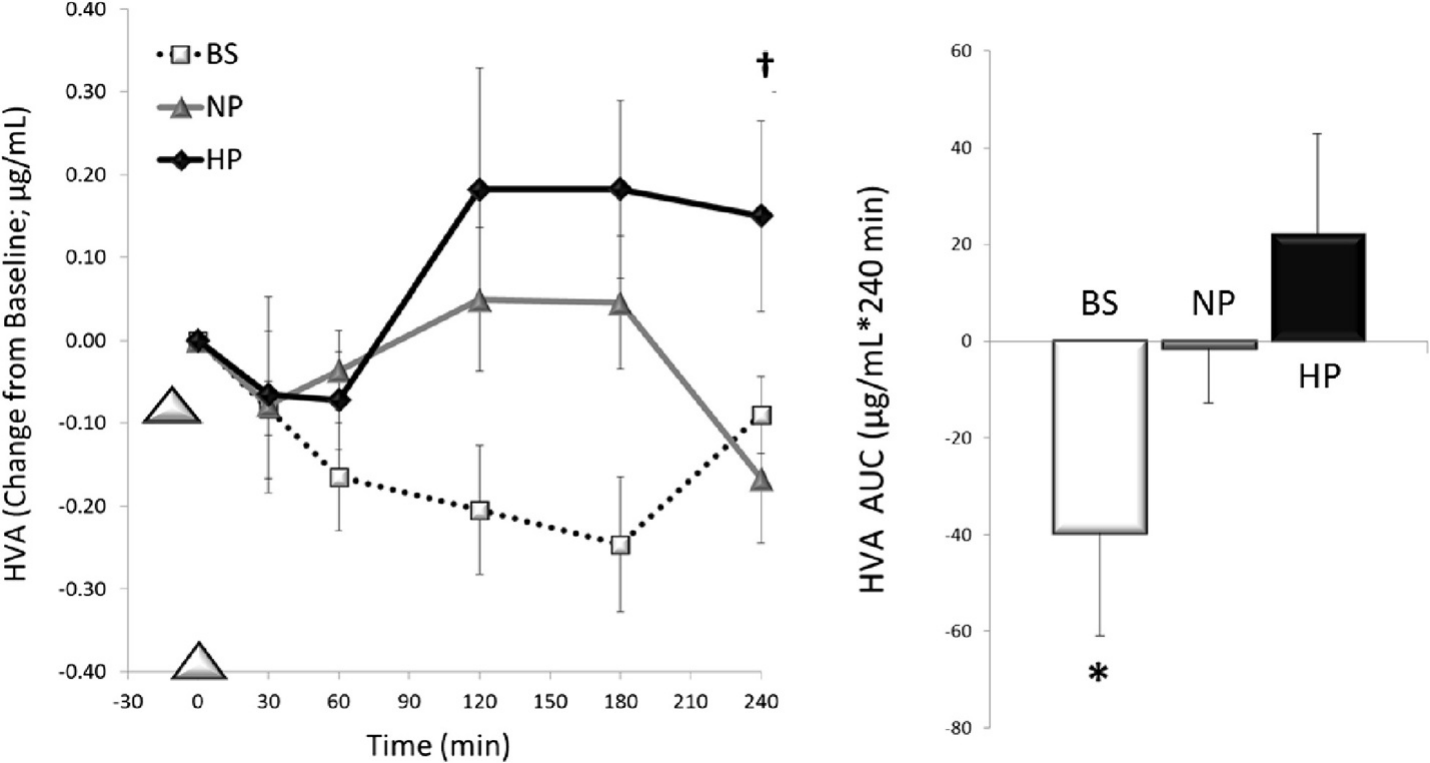
HVA responses over the 4 h post-breakfast period comparing breakfast skipping (BS), normal protein (NP), and high protein (HP) patterns; *Post-hoc analyses, BS vs. NP & HP, p < 0.05; †NP vs. HP, p = 0.09; Data is expressed as mean ± SEM.

As shown in Figures 1 and 2, the line graphs illustrate the responses throughout the Breakfast Skipping (BS), Higher Protein (HP), and Normal Protein (NP) testing days; the bar graphs depict the 4-hour AUC analyses across this period.

Main effects of time (P < 0.001), treatment (P < 0.01), and a time x treatment interaction (P < 0.01) were detected for the post-prandial sweet craving responses. The consumption of breakfast, regardless of type, led to an initial decline in sweet cravings followed by a progressive increase throughout the morning, whereas breakfast skipping led to a progressive increase over the 4 h period. When examining the 4 h AUC responses, the consumption of the NP and HP breakfast meals led to reduced 4-h AUC for cravings for sweet foods (NP: -1732 ± 848 mm*240 min; HP: -2949 ± 995 mm*240 min) vs. BS (798 ± 689 mm*240 min, both p < 0.05; Figure 1a)). No significant differences were observed between meals.

Main effects of time (P < 0.001), treatment (P < 0.01), and a time x treatment interaction (P < 0.01) were detected for the post-prandial cravings for savory foods. The consumption of breakfast, regardless of type, led to an initial decline in savory cravings followed by a progressive increase throughout the morning, whereas breakfast skipping led to a progressive increase over the 4 h period. When examining the 4 h AUC responses, the consumption of both breakfast meals led to reductions in 4-h cravings of savory foods (NP: -93 ± 766 mm*240 min; HP: -3185 ± 867 mm*240 min) vs. BS (2550 ± 954 mm*240 min, p = 0.07 and p < 0.01, respectively). The HP breakfast tended to produce reduced 4-h AUC for savory cravings vs. NP (p = 0.08).

Although no main effect of time (OP, 0.28) or treatment (OP, 0.48) was observed with post-prandial HVA concentrations, a time x treatment interaction was detected (p < 0.01, OP, 0.90). Specifically, only the HP breakfast (21.97 ± 22.66 ug/mL*240 min) led to greater 4-h HVA-AUC vs. BS (-39.64 ± 15.4 ug/mL*240 min, p < 0.05), whereas the NP breakfast did not (-1.48 ± 12.14 ug/mL*240 min, NS; Figure 2). Although the 4-h HVA-AUC was not different between breakfast meals, the HP meal tended to elicit greater pre-lunch (i.e. +240 min) HVA concentrations vs. NP (p = 0.09).

### Pearson’s correlational analyses

Pearson’s correlational analyses revealed that 4-h HVA AUC was associated with total protein content (r: 0.340; p < 0.03) and tyrosine content of the meal (r: 0.344; p < 0.03). However, no other associations were detected.

## Discussion

The addition of breakfast led to reductions in food cravings which were accompanied by increases in Homovanillic Acid concentrations, with slight improvements observed following the higher vs. normal protein meals. Collectively, these data suggest that the daily addition of breakfast, particularly one rich in protein, might serve as a beneficial strategy to reduce food cravings and modulate food reward in overweight/obese young people.

Much of the animal and human work concerning the dietary components that stimulate food reward, food cravings, and/or drive food preferences have centered around highly palatable foods which typically consist of high fat and high sugar foods. These energy dense, yet nutrient-void foods can lead to increased appetite, overeating, and weight gain over the long term [35]. Thus, identifying equally palatable, but nutrient-rich, healthier replacements might reduce some of these negative effects.

Dietary protein has been well documented to improve appetite control and food intake regulation through physiological increases in satiety and subsequent reductions in intake [24]. Although dietary fats and sugars are generally thought to be more rewarding and craved compared to other foods, similar cravings have been shown for protein-rich foods [36]. Further, as discussed in the review by Journel, et al. [37], dietary protein has also been proposed to modulate food hedonics. However, the mechanism of action by which the consumption of dietary protein elicits these responses remains unclear but has been postulated to be involved with dopamine production. Specifically, the rate of dopamine synthesis is sensitive to local substrate concentrations, primarily the amino acid tyrosine, which is influenced by the protein content of a single meal as well as within the overall diet [38]. Since tyrosine is the substrate required in the rate-limiting step of dopamine synthesis [38, 39], the consumption of higher protein meals containing increased tyrosine potentially leads to increases in dopamine synthesis. This has been demonstrated in an animal study in which rats fed a higher protein diet exhibited a substantial increase in central tyrosine which was accompanied by an increase in dopamine synthesis compared to rats fed lower protein diets [40]. In the current study, we showed that HVA concentrations were higher following the higher protein meal and were related to protein content and tyrosine content of the breakfast meal. Together, these data further support that dietary protein might be an important regulator of both physiological and hedonic food intake.

Another dietary factor which has significant effects on both aspects of food intake regulation is the common practice of skipping breakfast. In a pilot study from our lab, we showed that breakfast skipping adolescents exhibit greater appetite and reduced satiety throughout the morning, leading to increased energy intake; however, the addition of breakfast, particularly breakfast meals containing increased dietary protein, reversed these outcomes [9]. In a subsequent pilot study, we incorporated functional MRI to identify the neural responses to visual food stimuli prior to lunch in overweight/obese ‘breakfast skipping’ teen girls [41]. We found that breakfast skipping led to increased neural activation in brain regions controlling food motivation and food reward (i.e., hippocampus, amygdala, anterior cingulate, and parahippocampus) prior to lunch; however, the addition of a protein-rich breakfast led to reduced activation in these regions. The current study extended our previous fMRI findings to include HVA concentrations which serve as another marker of central food motivation and reward. We found that the addition of breakfast led to increased HVA concentrations throughout the morning with a trend for greater increases in HVA following the high protein breakfast vs. normal protein version. These data, along with the positive correlation between HVA concentrations and breakfast protein quantity, suggest that the consumption of increased dietary protein potentially stimulates the formation, secretion, and/or utilization of dopamine. The increased HVA concentrations throughout the morning were also accompanied by reduced food cravings, further supporting the role of dopamine on food reward.

Although central dopamine regulates a number of pathways in the body that impact cognition, motor control, mood, pain perception, and sexual behavior [42], it has also been shown to mediate food motivation and reward through a variety of learning and motivational pathway [43]. See Meye & Adan review [44]. Although dopamine is typically secreted in response to high fat foods, chronic exposure to these foods, particularly in obese individuals, leads to neural adaptations including reductions in dopamine receptor expression and decreased dopamine secretion [15]. In diet-induced obese rodents, this reduced dopamine response leads to an overcompensation of foods high in fat to potentially re-establish normal dopamine concentrations [45]. However, unlike dietary fat, the chronic consumption of protein-rich foods appears to elicit a more balanced, sustain influence on food reward as illustrated by the reductions in high fat and high sugar, evening snacking following the chronic (i.e., 7-d) consumption of high protein vs. normal protein breakfast meals [10].

In humans, psychostimulant drugs such as amphetamines and cocaine increase dopamine secretions and are known to have anorexigenic effects [46]. Further, the administration of dopamine agonists such as bromocriptine and methylphenidate have been shown to significantly reduce body fat and body weight in obese humans [47, 48]. Although the exact mechanisms behind this phenomenon have not been determined, these studies report reduced daily energy intake with the administrations of these agonists. Taken together, these data suggest that dopamine appears to play a critical role in modulating the reinforcing value and reward of food. Further, the dopamine pathway is blunted in obesity due to chronic exposure to highly palatable foods but can be re-established with pharmaceutical agents and potentially dietary factors including breakfast and increased dietary protein.

### Limitations

This study was likely to have been limited by the small sample size (n = 16). Although this sample size was adequate to detect differences between skipping breakfast vs. breakfast, a larger sample may have led to an increased ability to detect differences, particularly in HVA concentrations, between breakfast meals. However, further research, involving increased sample sizes, longer testing durations, and assessments of subsequent food intake are key in assessing the role of increased dietary protein at breakfast on dopamine, food motivation, and reward in overweight/obese teens.

## Conclusion

In conclusion, the addition of breakfast led to reductions in food cravings and increases in homovanillic acid, an index of central dopamine production, with the high protein breakfast eliciting greater responses. These data suggest that the daily addition of a breakfast, particularly one rich in protein, might be an important dietary strategy to reduce food cravings and potentially modulate the underlying substrates that control food hedonics/reward in overweight/obese young people.
